# Atypical Presentation of Sporadic Creutzfeldt–Jakob Disease in a 59-Year-Old Male Patient

**DOI:** 10.1192/bjo.2025.10732

**Published:** 2025-06-20

**Authors:** Ruzaika Jaufer

**Affiliations:** Norfolk and Suffolk NHS Foundation Trust, Norwich, United Kingdom

## Abstract

**Aims:** Creutzfeldt–Jakob Disease (CJD) is a rare, fatal neurodegenerative disorder caused by prion proteins, leading to progressive brain damage. CJD has sporadic, variant, genetic, and iatrogenic forms, with sporadic being the most common, affecting 1–2 people per million annually. It typically presents with rapid cognitive decline, motor dysfunction, and personality changes, with no effective treatment available.

**Methods:** A 59-year-old physically fit, male with no past medical history presented to A&E with a two-week history of behavioural changes, aggression, left facial droop, disorientation, poor coordination, and mild dysarthria. He had repetitive speech and his GCS on admission was 14/15.

Stroke was suspected and CT scan showed hypoattenuation of the right frontal lobe. Contrast MRI showed multifocal areas of gyriform diffusion restriction affecting bilateral cerebral hemispheres. Initial differential diagnoses were stroke, vasogenic oedema, old brain injury, post-ictal changes, encephalitis.

EEG and initial blood tests were normal. CSF analysis revealed elevated proteins, normal glucose and lactate and no oligoclonal bands. CSF virology and cultures were negative. A protein assay from specialist services in Edinburgh confirmed a diagnosis of sporadic CJD.

The patient was discharged to a care home with clonazepam and a plan to introduce low-dose quetiapine if needed. Upon transfer, he became increasingly agitated, displaying verbal and physical aggression towards staff and residents, wandering and damaging property. His sleep was poor, and his aggression unpredictable, escalating late in the evening.

The care home implemented a low-stimulus environment with one-to-one care and covert medication administration. Initially treated with quetiapine and benzodiazepines, his aggression persisted, prompting introduction of haloperidol, which resulted in some reduction in aggression. He was later transferred to an inpatient ward.

On the ward, his dysphasia worsened, but he had no further behavioural challenges. He received regular physiotherapy, occupational therapy and specialised nursing care. Parkinsonism symptoms prompted the discontinuation of haloperidol, which was later reinstated at a lower dose due to return of restlessness. Quetiapine was gradually tapered off. At discharge, he was compliant with medication, required assistance with feeding and personal care, had severe dysphasia and some ataxia.

**Results:** This case demonstrates the atypical presentation of CJD with neuropsychiatric symptoms at onset, rather than the usual neurological signs. The patient’s agitation and aggression required careful pharmacological adjustments and highlighted challenges in managing CJD’s neuropsychiatric symptoms.

**Conclusion:** This case highlights the importance of considering CJD in the differential diagnosis for patients with rapid-onset neuropsychiatric disturbances. Early recognition and symptomatic management, although not curative, can improve the patient’s quality of life during the disease’s progression.

